# Empirical vs. Susceptibility-Guided Treatment of *Helicobacter pylori* Infection: A Systematic Review and Meta-Analysis

**DOI:** 10.3389/fmicb.2022.913436

**Published:** 2022-06-14

**Authors:** Olga P. Nyssen, Marta Espada, Javier P. Gisbert

**Affiliations:** ^1^Gastroenterology Unit, Instituto de Investigación Sanitaria Princesa (IIS-Princesa), Hospital Universitario de La Princesa, Madrid, Spain; ^2^Universidad Autónoma de Madrid (UAM), Madrid, Spain; ^3^Centro de Investigación Biomédica en Red de Enfermedades Hepáticas y Digestivas (CIBEREHD), Madrid, Spain

**Keywords:** *Helicobacter pylori*, culture, tailored, susceptibility, empirical

## Abstract

**Background:**

Treating *Helicobacter pylori* infection according to antibiotic resistance has been frequently recommended. However, information on its real effectiveness is scarce.

**Aim:**

The aim of this study is to perform a meta-analysis comparing empirical vs. susceptibility-guided treatment of *H. pylori*.

**Methods:**

*Selection of studies*: Studies comparing empirical versus susceptibility-guided treatment were selected. *Search strategy*: electronic and manual up to August 2021. *Data synthesis*: by intention-to-treat (random-effects model).

**Results:**

Overall, 54 studies were included (6,705 patients in the susceptibility-guided group and 7,895 in the empirical group). *H. pylori* eradication rate was 86 vs. 76%, respectively (RR: 1.12; 95% CI: 1.08–1.17; *I*^2^: 83%). Similar results were found when only RCTs were evaluated (24 studies; RR: 1.16; 95% CI: 1.11–1.22; *I*^2^: 71%) and when susceptibility testing was assessed by culture (RR: 1.12; 95% CI: 1.06–1.18) or PCR (RR: 1.14; 95% CI: 1.05–1.23). For first-line treatments (naïve patients; 30 studies), better efficacy results were obtained with the susceptibility-guided strategy (RR: 1.15; 95% CI: 1.11–1.20; *I*^2^: 79%). However, for empirical first-line quadruple regimens, in particular (both with and without bismuth, excluding the suboptimal triple therapies), not based on CYP2C19 gene polymorphism, no differences in efficacy were found compared with the susceptibility-guided group (RR: 1.04; 95% CI: 0.99–1.09); this lack of difference was confirmed in RCTs (RR: 1.05; 95% CI: 0.99–1.12). For rescue therapies (13 studies, most 2^nd^-line), similar results were demonstrated for both strategies, including all studies (RR: 1.09; 95% CI: 0.97–1.22; *I*^2^: 82%) and when only RCTs were considered (RR: 1.15; 95% CI: 0.97–1.36).

**Conclusion:**

The benefit of susceptibility-guided treatment over empirical treatment of *H. pylori* infection could not be demonstrated, either in first-line (if the most updated quadruple regimens are prescribed) or in rescue therapies.

## Introduction

*Helicobacter pylori* (*H. pylori*) infection affects billions of people worldwide, which is the main cause of gastritis, peptic ulcer disease, and gastric cancer (Hooi et al., 2017). However, after more than 30 years of experience in the management of this infection, the ideal treatment regimen remains undefined.

Antibiotic resistance has been identified as the major factor affecting our ability to cure *H. pylori* infection, and the rate of resistance to several antibiotics—mainly clarithromycin—is steadily increasing in many geographic areas (Dore et al., 2000; Megraud et al., 2013; Camargo et al., 2014; Thung et al., 2016). A recent systematic review and meta-analysis assessed the distribution of *H. pylori* resistance to commonly used antibiotics in 65 countries and found that primary resistance rates to clarithromycin, metronidazole, and levofloxacin were ≥15% in most regions. Furthermore, increasing antibiotic resistance was observed in most countries (Savoldi et al., 2018). Accordingly, the World Health Organization (WHO) has designated clarithromycin-resistant *H. pylori* a high priority for antibiotic research and development.

Since antibiotic resistance is an evolving process, it seems mandatory to carry out point prevalence surveys on a regular basis to guide clinicians in their therapeutic choice (Megraud et al., 2013). A strategy that has been suggested to increase the eradication rate is individualized treatment according to antibiotic susceptibility testing (personalized treatment). However, the true utility of culture—with consequent antibiotic susceptibility testing—and the moment when it must be performed (before the first treatment or only after eradication failure) are both controversial. Of note, *H. pylori* culture is time-consuming, not always available on a routine basis, offers quite low sensitivity, and implies the performance of an endoscopic exploration (Zullo et al., 2003; Gisbert, 2011). Furthermore, culture is relatively expensive, not because of the cost of the procedure *per se*, but mainly because of the costs of the associated endoscopy required to obtain biopsy specimens.

Although susceptibility-guided therapy is recommended by many *H. pylori* consensus reports, the number of studies evaluating this strategy is, however, quite limited, and the evidence available to date regarding when and in whom culture should be performed is surprisingly scant. Currently, most physicians treat *H. pylori* infection without relying on antimicrobial susceptibility testing to choose the best regimen (Gisbert, 2020).

Therefore, the present study aimed to perform a meta-analysis comparing empirical vs. susceptibility-guided treatment of *H. pylori* including both first-line and rescue regimens.

## Methods

### General Criteria for Considering Studies for This Review

Randomized, quasi-randomized, and non-randomized controlled trials were eligible for inclusion in this review, whereas case reports, letters, editorials, comments, and reviews were excluded. Full-text forms and abstracts of the articles selected (in each of the searches) were reviewed, and those dealing with the susceptibility-guided treatment of *H. pylori* infection were recorded and were eligible for inclusion. No restrictions by date of publication or by language were considered.

The studied population included adults or children diagnosed as positive for *H. pylori*. Patients could be treated with any of the available eradication treatments for *H. pylori* infection in any line of treatment. Trials had to compare the efficacy of an *H. pylori* eradication treatment based on a previous susceptibility-guided diagnostic test with that of empirical treatment. Pre-treatment diagnostic methods for *H. pylori* detection should comprise one or more of the most commonly validated tests: urea breath test, histology, rapid urease test, and stool antigen test; for susceptibility-guided treatments, studies should include methods to test antimicrobial susceptibility on gastric biopsies such as PCR or culture.

Eligible studies should include accessible data on successful eradication rates in both tailored and empirical groups.

### Outcome Measures

The primary endpoint was intention-to-treat efficacy (*H. pylori* eradication rate). Reported efficacy was considered as the rate (proportion) of patients cured among the total of treated patients. Trials were included if they reported the number of patients with *H. pylori* eradication in each treatment arm; otherwise, the numerator was calculated from the percentage of eradication reported and the intention-to-treat (ITT) sample size.

Trials were eligible if *H. pylori* eradication was confirmed using a rapid urease test, histology or culture of an endoscopic biopsy sample, or by a urea breath test or a monoclonal stool antigen test, at least 4 weeks after completion of treatment. Trials, in which only serology test was performed, were excluded.

### Search Methods for Identification of Studies

#### Search Strategy

Bibliographical searches were performed in the MEDLINE, EMBASE, and the Cochrane Library electronic databases up to August 2021 based on the following words (all fields): pylori AND [(culture OR culture-based OR culture-guided OR tailored OR susceptibility OR susceptibility-guided OR “antimicrobial susceptibility” OR “susceptibility testing”) OR (empiric OR empirical)].

Reference lists of the articles selected by electronic searching were examined in detail to further identify relevant studies. In addition, references of articles retrieved, significant reviews, and the personal databases of the authors were also checked for eligible publications.

### Data Collection and Analysis

#### Selection of Studies

The Preferred Reporting Items for Systematic reviews and Meta-Analyses (PRISMA) approach (www.prismastatement.org) was used to develop a diagram to schematize the different steps of study selection (Liberati et al., 2009; Page et al., 2021).

Before the selection of studies, duplicates were removed in the citation manager. The selection of studies was conducted in two phases: a first screening of titles and abstracts to identify potentially relevant citations; and a second phase, where full texts of the previously selected studies were retrieved. Selection criteria were applied to full texts for definite inclusion. Two reviewers (OPN and ME) performed the screenings independently; disagreements were resolved by consensus with a third reviewer (JPG). The reason for the exclusion of a given study was reported in the second phase only as appropriate.

### Data Extraction

A pre-tested data extraction form was used in a pilot test before the final collection of data to test its reliability. The following information was extracted from each study: first author; year of publication of the study; country; population (adult or children); study design (RCT or non-RCT); treatment line; susceptibility test; clarithromycin resistance rate (%); metronidazole resistance rate (%); levofloxacin resistance rate (%); type of empirical regimen; eradication rate with the empirical regimen; and eradication rate with the susceptibility-guided regimen. Two reviewers (OPN and ME) performed the data extraction independently; disagreements were resolved by consensus with a third reviewer (JPG).

### Assessment of the Risk of Bias in Included Studies

The risk of bias was assessed independently by two reviewers (OPN and ME); disagreements were resolved by consensus with a third reviewer (JPG) in accordance with the Cochrane Collaboration's current recommendations (Higgins et al., 2009).

For RCTs, the Cochrane Risk of Bias (RoB) tool was used and the six quality items were evaluated: random sequence generation (selection bias), allocation concealment (selection bias), blinding of participants and personnel (performance bias), blinding of outcome assessment (detection bias), incomplete outcome data (attrition bias), and selective reporting (reporting bias). A study was considered to be an RCT if it was explicitly described as “randomized.” This should include the use of words such as “random,” “randomly,” or “randomization.” We then rated the potential randomized trial as truly random, pseudo-random (randomization was mentioned but the method used was not reported), or non-random, based on the definitions by the Cochrane Handbook (Higgins et al., 2009).

For quasi-randomized trials (that is, non-random but controlled studies) and non-RCTs, the RoB criteria for EPOC Reviews (Guide for review authors on assessing study quality) advocated by the Cochrane was used. The same quality domains (as for RCTs) were assessed, but that related to the evaluation of randomization was reported as “high risk of bias” as no allocation of the sequence was generated as per the study design.

### Assessment of Heterogeneity

The possible sources of diversity in the trial's characteristics were evaluated. We performed the Chi^2^ test for heterogeneity for each combined analysis, where P < 0.10 indicated significant heterogeneity between studies (Higgins and Thompson, 2002). Graphical methods (forest plots) were also used to complete the Chi^2^ test assessment.

The *I*^2^ statistic was used to assess the heterogeneity of the studies, following the recommendation of the Cochrane Collaboration's Handbook for Systematic Reviews of Interventions (Higgins et al., 2009), as follows: 0 to 40%, unimportant heterogeneity; 40 to 75%, moderate heterogeneity; 75 to 100% considerable heterogeneity.

### Assessment of Reporting Biases

To assess publication bias, funnel plot asymmetry was inspected visually by examining the relationship between the treatment effects and the standard error of the estimate.

### Data Synthesis

To collate, combine, and summarize the information obtained, a quantitative approach was undertaken. The evidence collected in the included studies was synthesized by summarizing the information related to the effect size of all studies, for each comparison and for each subgroup analysis. A meta-analysis was therefore performed combining the calculated risk ratios (RRs) of the individual studies with their corresponding 95% confidence intervals (CIs), using a random effects model (Mantel–Haenszel). Additional sensitivity analyses were performed to check the robustness of the results (DerSimonian and Laird, 1986; Egger et al., 1997).

The subgroup analyses were pre-planned to explore the possible sources of heterogeneity according to the study design (RCT vs. non-RCT), treatment line (naïve vs. rescue), susceptibility testing (culture vs. PCR), RCT by treatment line, and RCT by susceptibility test. The last group evaluating the empirical first-line quadruple treatments only was also included, to perform the most equitable comparison according to the most updated recommended first empirical quadruple treatments (i.e., non-bismuth and bismuth quadruple therapy) (Malfertheiner et al., 2017).

Analyses were performed using the freeware program Review Manager (RevMan) version 5.4.1 (2020).

## Results

### Description of Studies

In total, 17,383 citations were retrieved from the following electronic databases: PubMed and EMBASE, up to August 2021. After removing duplicates, a total of 13,941 citations were screened. After reviewing the abstracts and full texts, 47 studies (Romano et al., 2000, 2003; Toracchio et al., 2000; Avidan et al., 2001; Street et al., 2001; Lamouliatte et al., 2003; Miwa et al., 2003; Neri et al., 2003; Marzio et al., 2006; Yahav et al., 2006; Furuta et al., 2007; Kawai et al., 2008; Wang et al., 2008; Zhou et al., 2010, 2016; Bontems et al., 2011; Molina-Infante et al., 2012; Cosme et al., 2013, 2016; Lee et al., 2013; Martos et al., 2014; Park et al., 2014; Dong et al., 2015; Zhuo et al., 2015; Kwon et al., 2016; Miyaki et al., 2016; Ferenc et al., 2017; Gweon et al., 2018; Huang et al., 2018; Liou et al., 2018; Mascellino et al., 2018; Tanabe et al., 2018; Byambajav et al., 2019; Chen et al., 2019; Cho et al., 2019; Choi et al., 2019, 2021; Delchier et al., 2019; Ong et al., 2019; Pan et al., 2020; Saracino et al., 2020; Zhang et al., 2020; Bonoso Criado et al., 2021; Cha et al., 2021; Chang et al., 2021; Choe et al., 2021) were finally included in the present systematic review. A total of 54 different treatment comparisons were evaluated both in the quantitative and qualitative synthesis since some studies assessed more than one treatment comparison.

**Figure 1:**
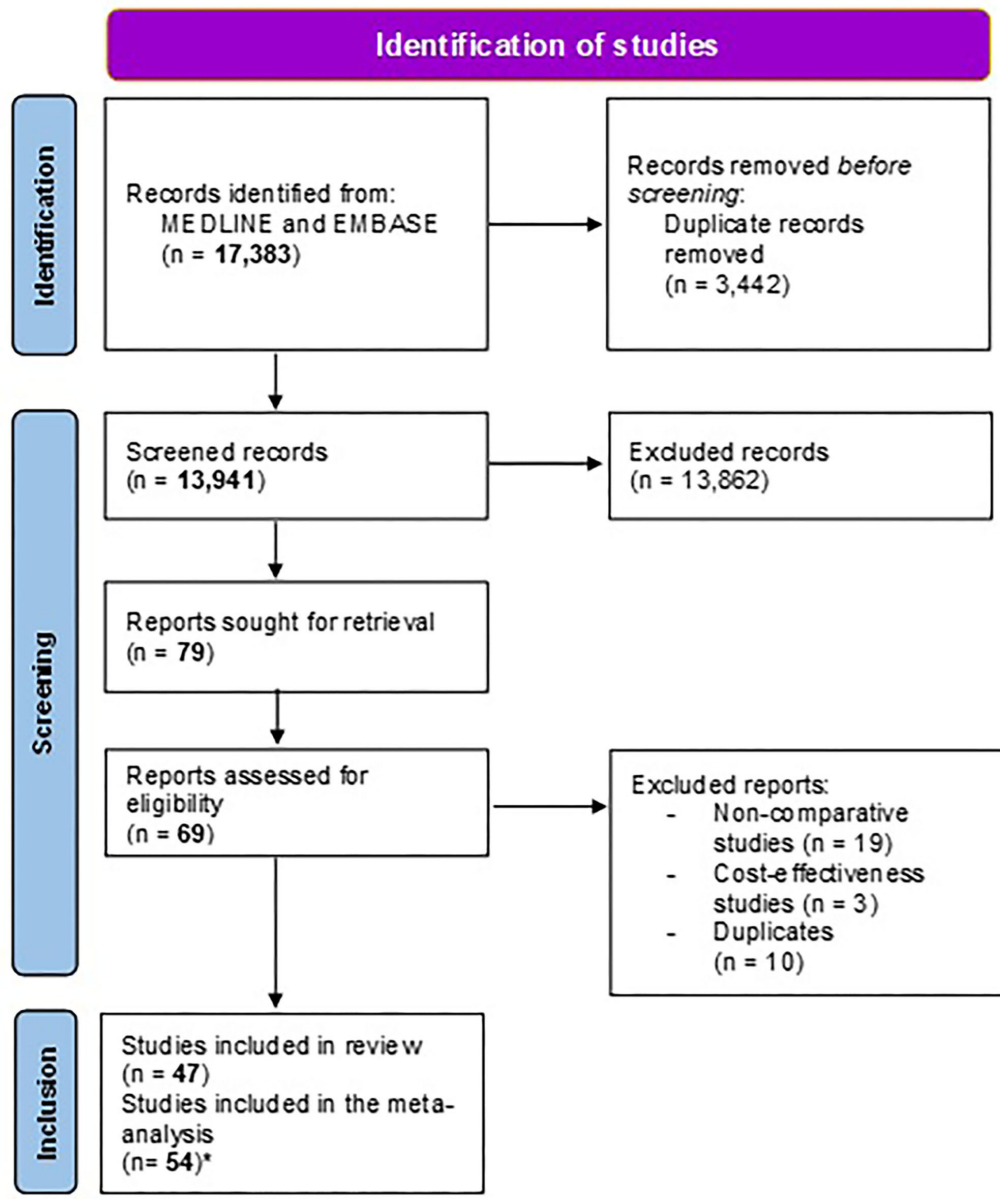
Study flow chart. ^*^The number of studies included in the meta-analysis is higher than the number of studies included in the systematic review because certain studies included different treatment groups that were meta-analyzed separately (i.e., we considered as many study groups/comparisons as treatment arms).

The different steps in the selection of studies and the reasons for the exclusion of studies [non-comparative (Gasbarrini et al., 2000; Gomollon et al., 2000; Chi et al., 2003; Cammarota et al., 2004; Fiorini et al., 2013; Liou et al., 2013; Choi et al., 2014; Sugimoto et al., 2014; Draeger et al., 2015; Stamboliyska et al., 2015; Han et al., 2016; Cosme et al., 2017, 2019; Costa et al., 2017; Králícek et al., 2017; Blümel et al., 2018; Kwon et al., 2019; Lee et al., 2019; Yu et al., 2019; Zhu and Wu, 2019) and cost-effectiveness (Breuer and Graham, 1999; Qasim et al., 2004; Faber et al., 2005; Cammarota et al., 2014)] are reported in the PRISMA flow chart (Figure 1).

The search time span was from the year 2000 to 2021, and published studies were mainly from Asian and European countries. All studies were on adults except for two, which evaluated children. One of them was an RCT (Bontems et al., 2011) and the other one was a prospective observational cohort (Zhang et al., 2020), and both used culture for the diagnosis of the infection and tailoring of the treatment.

We found 31 RCTs and 23 non-RCTs. In 30 studies, patients were naïve to treatment and the majority assessed patients treated with a second-line rescue therapy, except for three studies (Bontems et al., 2011; Huang et al., 2018; Liou et al., 2018), in which patients were treated with a third-line therapy. Baseline characteristics, diagnostic methods, and prescribed treatments are reported in Table 1. Most studies are tested for clarithromycin resistance. In four studies, (Miwa et al., 2003; Furuta et al., 2007; Zhou et al., 2016; Zhang et al., 2020) patients received a tailored therapy based on CYP2C19 polymorphism.

**Table 1:** Characteristics of studies comparing empirical vs. susceptibility-guided treatment for *H. pylori* infection.

**Author**	**Year**	**Country**	**Population**	**Design**	**Treatment** **line**	**Susceptibility** **test**	**C res** **(%)**	**M res** **(%)**	**L res** **(%)**	**Empirical** **regimen**	**Eradication rate with empiric regimen**	**Eradication rate with susceptibility-guided regimen**
Toracchio et al. (2000)	2000	Italy	Adults	R	1^st^	Agar dilution	13	33	-	PPI-C-M, 10 d	42/56 (75%)	48/53 (91%)
Romano et al. (2000)	2000	Italy	Adults	R	1^st^	E-test	12	22	-	PPI-C-M, 7 d	31/40 (77%)	38/40 (95%)
Street et al. (2001)	2001	Italy	Adults	NR	1^st^	E-test	16	56	-	PPI/Ra-A-C, 8 d	61/75 (81%)	62/63 (98%)
Avidan et al. (2001)	2001	Israel	Adults	R	2^nd^	E-test	100	0	-	PPI-A-C, 10 d	5/5 (100%)	5/5 (100%)
Miwa et al. (2003)	2003	Japan	Adults	R	2^nd^	Dry plate	71	-	-	PPI-A-M, 10 d	36/39 (92%)	31/38 (82%)^*^
Neri et al. (2003)	2003	Italy	Adults	R	1^st^	E-test	7	12	-	PPI-A-C, 7 d RBC-C-M, 7 d	78/116 (67%)	88/116 (76%)
Romano et al. (2003)	2003	Italy	Adults	R	1^st^	E-test	12	22	-	PPI-C-M, 7 d	58/75 (77%)	71/75 (95%)
Lamouliatte et al. (2003)	2003	France	Adults	R	2^nd^	E-test	64	53	-	PPI-A-C, 7 d PPI-A-C, 14 d PPI-A-M, 14 d	83/172 (48%)	84/113 (74%)
Marzio et al. (2006)	2006	Italy	Adults	R	1^st^	Agar dilution	22	32	10	PPI-A-L, 10 d	36/39 (92%)	39/41 (95%)
Marzio et al. (2006)	2006	Italy	Adults	R	2^nd^	Agar dilution	43	70	12	PPI-A-L, 10 d	26/32 (81%)	50/51 (98%)
Yahav et al. (2006)	2006	Israel	Adults	NR	2^nd^	E-test	59	47	-	PPI-A-C, 7 d PPI-A-M, 7 d PPI-B-T-M, 7 d	31/49 (63%)	42/49 (86%)
Furuta et al. (2007)	2007	Japan	Adults	R	1^st^	PCR	30	-	-	PPI-A-C, 7 d	105/150 (70%)	144/150 (96%)^*^
Kawai et al. (2008)	2018	Japan	Adults	R	1^st^	PCR (stools)	48	-	-	PPI-A-C, 7 d	25/35 (71%)	33/35 (94%)
Wang et al. (2008)	2008	China	Adults	R	1^st^	Culture	10	-	-	PPI-A-C, 7 d PPI-C-M, 7 d	57/80 (71%)	36/40 (90%)
Zhou et al. (2010)	2010	China	Adults	R	1^st^	Agar dilution	15	-	-	PPI-C-M, 10 d	107/135 (79%)	117/125 (94%)
Bontems et al. (2011)	2011	Brussels, Italy, France	Children	R	1^st^ and ≥2^nd^	E-test	16	20	-	PPI-A, 5 d + PPI-C-M, 5 d	68/83 (82%)	59/82 (72%)
Molina-Infante et al. (2012)	2012	Spain	Adults	NR	1^st^	E-test	20	34	-	PPI-A-C-M, 10 d	182/209 (87%)	70/87 (80%)
Lee et al. (2013)	2013	Korea	Adults	NR	1^st^	PCR	22	-	-	PPI-A-C, 7 d PPI-C-M, 7 d	433/616 (70%)	176/218 (81%)
Cosme et al. (2013)	2013	Spain	Adults	NR	1^st^	E-test	13	-	-	PPI-A-C, 10 d	51/104 (49%)	113/134 (84%)
Park et al. (2014)	2014	Korea	Adults	R	1^st^	Agar dilution	25	46	37	PPI-A-C, 7 d	41/57 (72%)	54/57 (95%
Martos et al. (2014)	2014	Spain	Adults	R	1^st^	E-test	9	-	-	PPI+A+C, 10 d	36/54 (67%)	52/55 (94%)
Zhuo et al. (2015)	2015	China	Adults	R	1^st^	Agar dilution	17	95	28	PPI-A-C-B, 14 d	405/500 (81%)	281/313 (90%)
Dong et al. (2015)	2015	China	Adults	R	1^st^	E-test, PCR	40	53	56	PPI-A-C-B, 14 d	33/45 (73%)	41/45 (91%)
Zhou et al. (2016)	2016	China	Adults	R	1^st^	E-test	49	66	-	PPI-A-C-B, 10 d PPI-A-C-M, 10 d	545/700 (78%)	282/318 (89%)^*^
Kwon et al. (2016)	2016	Korea	Adults	NR	2^nd^	Agar dilution	85	52	-	PPI-B-T-M, 14 d PPI-A-Mo, 14 d	130/178 (73%)	36/41 (88%)
Cosme et al. (2016)	2016	Spain	Adults	NR	1^st^	E-test	16	-	-	PPI-A-C-M, 10 d	103/118 (87%)	98/104 (94%)
Miyaki et al. (2016)	2016	Japan	Adults	NR	1^st^	Agar dilution	30	-	-	PPI-A-C, 7 d	101/132 (76%)	119/128 (93%)
Ferenc et al. (2017)	2017	Poland	Adults	NR	1^st^	E-test	55	57	6	PPI-A, 5 d + PPI-C-M, 5 d PPI-A-L, 14 d	26/30 (87%)	43/45 (95%)
Liou et al. (2018)	2018	Taiwan	Adults	R	≥3^rd^	PCR	92	69	70	PPI-A, 7 d + PPI-M-L/C/T, 7 d	12/20 (60%)	17/21 (81%)
Liou et al. (2018)	2018	Taiwan	Adults	R	≥3^rd^	PCR	93	66	60	PPI-A, 7 d + PPI-M-L/C/T, 7 d	148/205 (72%)	160/205 (78%)
Gweon et al. (2018)	2018	Korea	Adults	NR	1^st^	PCR	37	-	-	PPI-A-C, 7 d	230/319 (72%)	191/208 (92%)
Gweon et al. (2018)	2018	Korea	Adults	NR	2^nd^	PCR	37	-	-	PPI-B-T-M, 7 d	66/75 (88%)	8/9 (89%)
Huang et al. (2018)	2018	Taiwan	Adults	NR	3^rd^	E-test	75	67	95	PPI-A-T-M, 14 d	14/27 (52%)	35/43 (81%)
Mascellino et al. (2018)	2018	Italy	Adults	NR	≥2^nd^	E-test	50	68	-	PPI-B-T-M, 14 d PPI-A-L, PPI-A-R Other regimens	8/10 (80%)	20/30 (67%)
Tanabe et al. (2018)	2018	Japan	Adults	NR	1^st^	Agar dilution	23	4	-	PPI-A-C, 7 d	619/780 /79%)	198/212 (93%)
Ong et al. (2019)	2019	Korea	Adults	R	1^st^	PCR	26	-	-	PPI-A-C-M, 14 d	169/196 (86%)	164/201 (82%)
Chen et al. (2019)	2019	China	Adults	R	1^st^	Agar dilution	35	83	47	PPI-B-A-M, 14 d	82/96 (85%)	262/286 (92%)
Cho et al. (2019)	2019	Korea	Adults	NR	1^st^	PCR	23	-	-	PPI-A-M, 7 d	186/327 (57%)	115/150 (77%)
Choi et al. (2019)	2019	Korea	Adults	NR	1^st^	PCR	24	-	-	PPI-B-T-M, 14 d	98/104 (94%)	48/50 (96%)
Byambajav et al. (2019)	2019	Mongolia	Adults	NR	1^st^	Agar dilution	37	74	-	PPI-A-C, 10 d PPI-A-C-B, 10 d PPI-A, 5 d + PPI-C-M, 5 d	204/270 (75%)	41/46 (89%)
Delchier et al. (2019)	2019	France	Adults	R	1^st^	PCR	23	-	13	PPI-A-C, 7 d	152/208 (73%)	177/207 (85%)
Zhang et al. (2020)	2020	China	Children	NR	2^nd^	Culture	96	4	7	PPI-A-M-B	74/75 (99%)	46/64 (72%)^*^
Saracino et al. (2020)	2020	Italy	Adults	NR	≥2^nd^	E-test	83	67	47	PPI-Pylera^®^, 10 d	161/186 (87%)	875/1037 (84%)
Pan et al. (2020)	2020	China	Adults	R	1^st^	Agar dilution	67	86	64	PPI-A-C-B, 14 d	100/157 (64%)	238/310 (77%)
Ji et al. (2020)	2020	China	Adults	R	≥2^nd^	Agar dilution	67	98	51	PPI-A-L-B, 14 d PPI-A-F-B, 14 d	156/210 (74%)	164/210 (78%)
Bonoso Criado et al. (2021)	2021	Spain	Adults	R	1^st^	Culture	23	25	19	PPI-B-T-M, 10 d	43/45 (96%)	39/43 (91%)
Bonoso Criado et al. (2021)	2021	Spain	Adults	R	2^nd^	Culture	23	25	19	PPI-B-T-M, 10 d	6/6 (100%)	8/9 (89%)
Bonoso Criado et al. (2021)	2021	Spain	Adults	R	3^rd^	Culture	23	25	19	PPI-B-T-M, 10 d	2/4 (50%)	1/1 (100%)
Chang et al. (2021)	2021	Korea	Adults	NR	1^st^	PCR	32	-	-	PPI-A-C, 7d	183/198 (92%)	256/292 (88%)
Choe et al. (2021)	2021	Korea	Adults	NR	1^st^	PCR	-	-	-	PPI-A-C, 14d	22/27 (82%)	124/139 (89%)
Choe et al. (2021)	2021	Korea	Adults	NR	1^st^	PCR	-	-	-	PPI-A, 5 d + PPI-C-M, 5 d	91/111 (82%)	8/10 (80%)
Choe et al. (2021)	2021	Korea	Adults	NR	1^st^	PCR	-	-	-	PPI-B-T-M, 14 d	15/17 (88%)	55/60 (92%)
Choi et al. (2021)	2021	Korea	Adults	R	1^st^	PCR	26	-	-	PPI-A-C-M, 10 d	88/107 (82%)	91/110 (83%)
Cha et al. (2021)	2021	Korea	Adults	R	1^st^	PCR	22	-	-	PPI-B-T-M, 7 d	142/161 (88%)	118/147 (80%)

*R, randomized; NR, non-randomized; C res, resistance to clarithromycin; M res, resistance to metronidazole; L res, resistance to levofloxacin; PPI, proton pump inhibitor; A, amoxicillin; C, clarithromycin; M, metronidazole (or tinidazole); B, bismuth; T, tetracycline; L, levofloxacin; Mo, moxifloxacin; R, rifabutine; Ra, ranitidine; RBC, ranitidine bismuth citrate; Pylera^®^, single capsule containing bismuth, tetracycline, and metronidazole; -, information was not reported/available. Eradication rate was calculated by intention-to-treat analysis*.

* *The dose of PPI in the susceptibility-guided regimen was also based on CYP2C19 gene polymorphism*.

### Overall Results

From all studies, 14,600 patients were analyzed (6,705 patients in the susceptibility-guided group and 7,895 in the empirical group). Overall, *H. pylori* eradication was significantly better for the susceptibility-guided treatment than for the empirical treatment, 86 vs. 76%, respectively (RR: 1.12; 95% CI: 1.08–1.17; *I*^2^: 83%; Supplementary Figure 1).

Results of meta-analyses comparing the effectiveness of the empirical and the susceptibility-guided treatments between different groups (by treatment line, study design, tailored therapy, or recommended empirical quadruple therapy) are detailed below.

### Treatment Line

#### First-Line Therapy

A total of 35 studies were included in this analysis, with 10,894 patients treated with first-line treatment. Statistically significant differences were found in cure rates in favor of susceptibility-guided therapy (87%) vs. empirical treatment (78%); however, results were highly heterogeneous (RR: 1.13; 95%CI: 1.08, 1.17; *I*^2^: 83%; Supplementary Figure 2).

Sensitivity analyses confirmed that susceptibility-guided therapy was also superior to first-line clarithromycin-based triple therapy, in areas with high (i.e., over 20%) clarithromycin resistance (RR: 1.13; 95% CI: 1.03, 1.25; *I*^2^: 90%) and also in those with low clarithromycin resistance (RR: 1.24; 95% CI: 1.15, 1.32; *I*^2^: 45%).

#### Rescue Therapy

A total of 16 studies were on rescue (more than one treatment failure) therapy. When patients receiving a second- (1,131) or third-line (152) treatment were evaluated separately, no differences were found between groups. Likewise, when all rescue lines were grouped (from 2^nd^ to 3^rd^) and analyzed together (1,356 participants), no differences were reported (RR: 1.07; 95% CI: 0.97–1.18; *I*^2^: 78%, Supplementary Figure 2).

### Susceptibility Test

Similar results were reported when culture (36 studies; RR: 1.11; 95% CI: 1.05–1.16; *I*^2^: 83%) or PCR (16 studies; RR: 1.08; 95% CI: 1,01–1,16; *I*^2^: 84%; Supplementary Figure 3) was used as a method to test antibiotic susceptibility; in both cases, the efficacy of the susceptibility-guided treatment was higher than that of the empirical treatment (85 vs. 77% and 86 vs. 78%, respectively). Heterogeneity between groups was high (*I*^2^*:* 83%); however, no significant variation in the mean effects was found between the different subgroups (*P* = 0.64).

### Randomized Controlled Trials vs. Non-randomized Controlled Trials

In total, 27 RCTs (encompassing 31 comparisons) were included in the meta-analysis; that is, 7,325 patients (3,502 in the susceptibility-guided and 3,823 in the empirical treatment group) were evaluated. *H. pylori* eradication was achieved in 85% of patients in the susceptibility-guided group vs. 76% in the empirical group (RR: 1.13; 95% CI: 1.07–1.18; *I*^2^: 74%; Supplementary Figure 4). In this subgroup, one study (Bontems et al., 2011) was on children, nevertheless, excluding this study from the group did not vary the result of the sensitivity analysis. Heterogeneity was considerable in the RCT group, but lower than that of the overall assessment including all study designs (74 vs. 82%; respectively).

In non-RCTs, 8,000 patients (3,698 in the susceptibility-guided group and 4,302 in the empirical treatment group) were analyzed. In this sub-group, eradication was also higher in the susceptibility-guided group than in the empirical group (RR: 1.07; 95% CI: 1.01–1.14; *I*^2^: 86%). Likewise, the exclusion of one study (Zhang et al., 2020) on children did not vary the overall result of the sensitivity analysis. In addition, heterogeneity was significantly higher in the non-RCT group than when only RCTs were evaluated (86 vs. 74%, respectively; *p* < 0.001).

#### Randomized-Controlled Trials by Treatment Line

All the RCTs included could be meta-analyzed by treatment line except for the one by Bontems et al. (2011), in which eradication data could not be extracted separately for the first- and second-line treatment arms.

**Figure 2:**
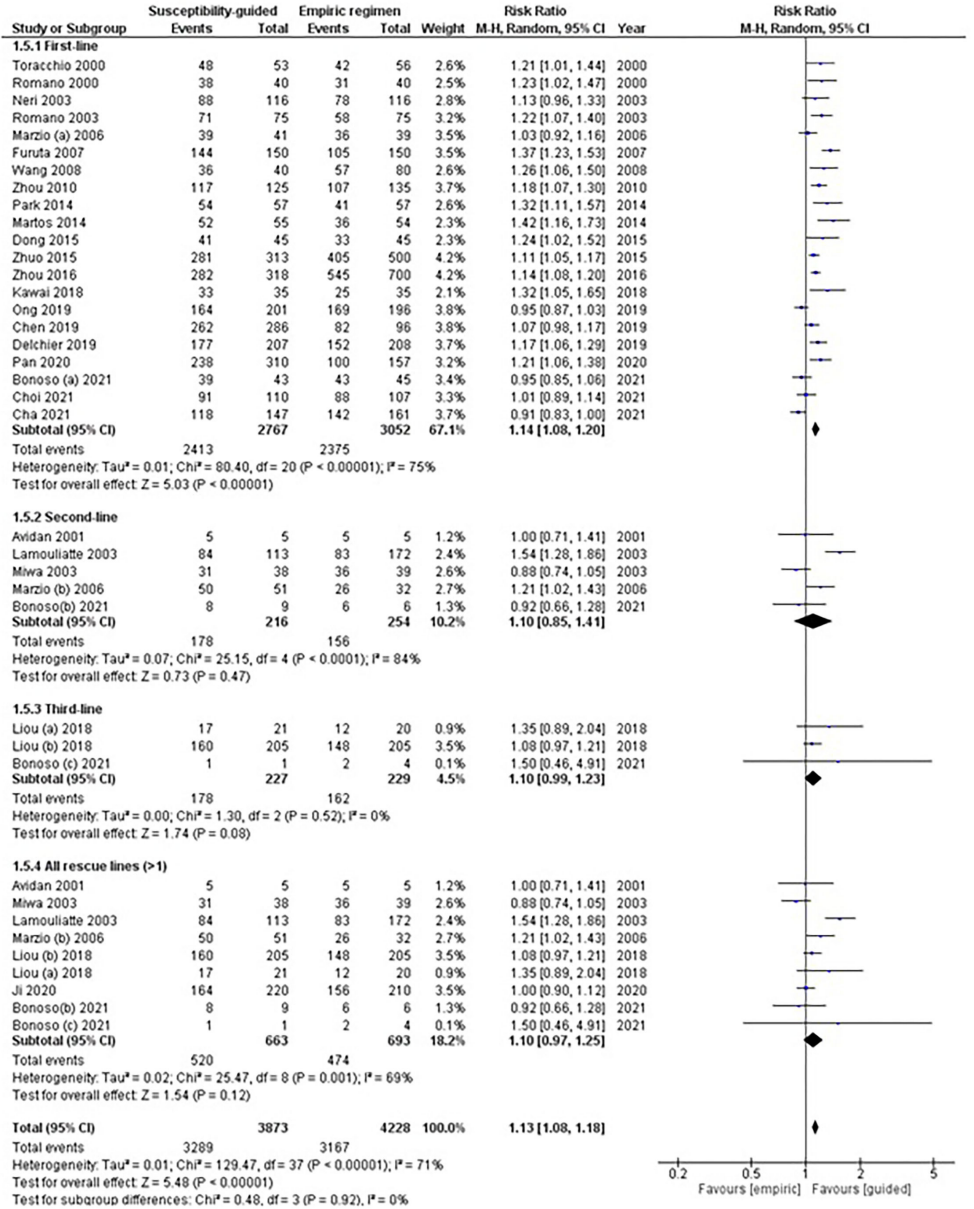
Forest plot of randomized controlled studies comparing the effectiveness of susceptibility-guided and empirical treatment according to treatment line. M-H, Mantel–Haenszel; CI, confidence interval.

In total, 21 comparisons were evaluated, where 5,819 naïve patients had been randomized to receive either a first-line empirical therapy or a susceptibility-guided treatment. Statistically significant differences were reported in eradication rates between groups (78 vs. 87%, respectively), with moderate heterogeneity between arms (RR: 1.14; 95% CI: 1.08–1.20; *I*^2^: 75%; Figure 2).

No statistical differences were observed in second- (RR: 1.10; 95% CI: 0.85–1.42; *I*^2^: 84%) or subsequent rescue treatment lines; that is, when participants received more than one eradication treatment (RR: 1.10; 95% CI: 0.97–1.25; *I*^2^: 69%). Two RCTs (Liou et al., 2018; Bonoso Criado et al., 2021) reported data on patients treated with a third-line treatment, with no differences between treatment arms.

#### Randomized-Controlled Trials by Susceptibility Test

A total of 24 RCTs used culture and 8 PCR-based methods to determine (on gastric biopsies only) any bacterial antibiotic resistance (Table 1). Overall data were reported with moderate to high heterogeneity for each of the subgroup analyses and the test for subgroup differences was reported not significant.

**Figure 3:**
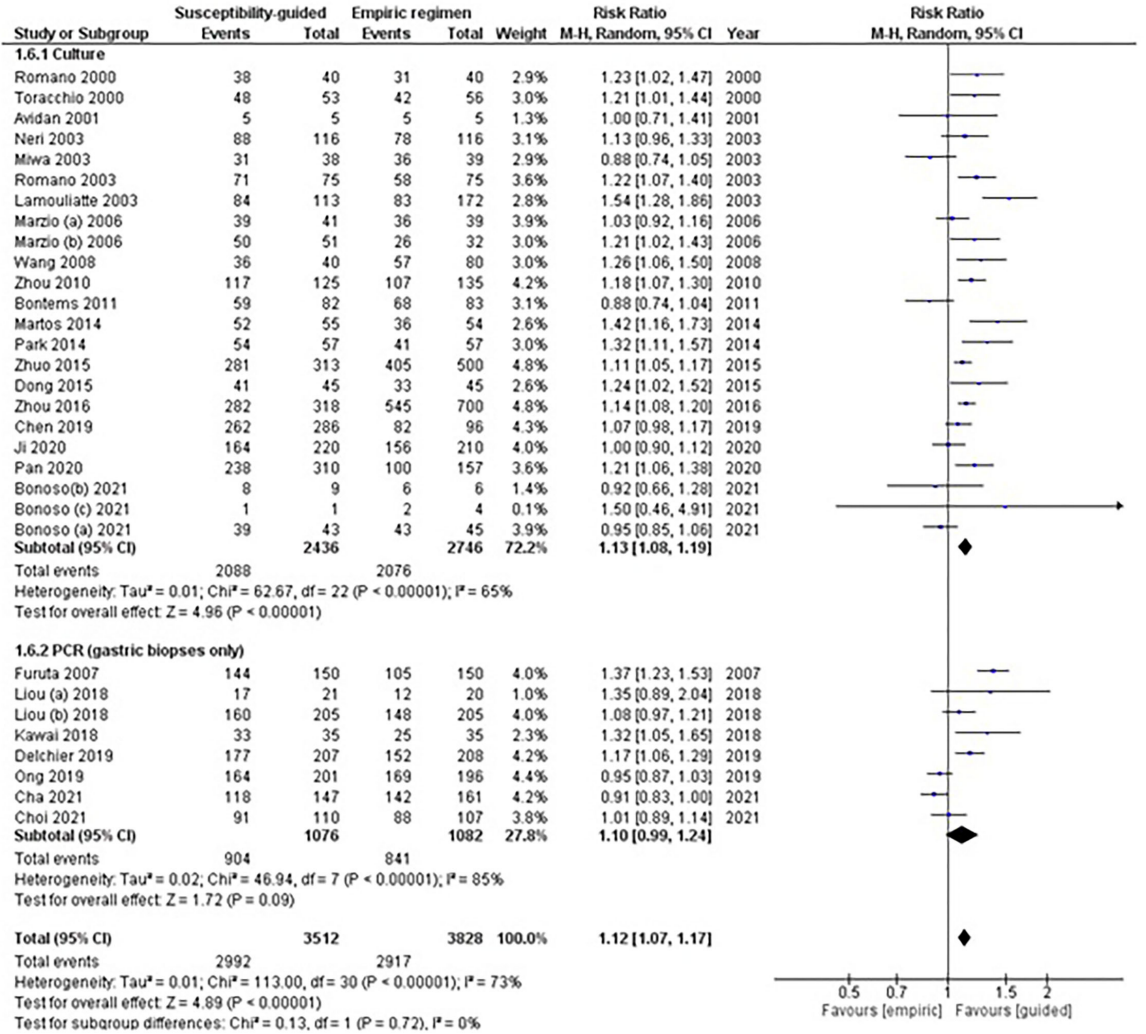
Forest plot of randomized controlled studies comparing the effectiveness of susceptibility-guided and empirical treatment according to susceptibility method (culture *vs*. polymerase chain reaction). M-H, Mantel–Haenszel; CI, confidence interval; PCR, polymerase chain reaction.

Among studies with culture testing, results were moderately heterogeneous (RR: 1.13, 95% CI: 1.08–1.19, *I*^2^: 65%; Figure 3), and eradication rates were statistically higher in the guided-treatment arm than in empirically treated participants (86 vs. 76%; respectively). In this same subgroup, sensitivity analyses performed among naïve patients showed similar results (RR: 1.13, 95% CI: 1.07–1.20, *I*^2^: 81%). However, when rescue treatments (more than one treatment failure) were considered, no statistically significant differences were found between treatment arms (RR: 1.05, 95% CI: 0.95–1.17, *I*^2^: 85%).

Among studies using PCR, no statistically significant differences were found between treatment groups (RR: 1.10, 95% CI: 0.99–1.24, *I*^2^: 85%). In this subgroup, sensitivity analyses performed among naïve patients showed similar results (RR: 1.10, 95% CI: 0.95–1.26, *I*^2^: 89%). One study (Liou et al., 2018) assessing third-line treatment, where two comparisons were available, showed no significant differences between treatments when data from both comparisons were pooled (RR: 1.10, 95% CI: 0.98–1.24, *I*^2^: 3%).

Two other studies (Kawai et al., 2008; Dong et al., 2015) were not included in this subgroup meta-analysis as they used PCR on stool, and both PCR and E-test, respectively, and results were not reported separately.

### Empirical First-Line Quadruple Treatment

The recommendations of the Consensus guidelines on *H. pylori* first-line therapy were used to select studies for this subgroup meta-analysis. Only those studies evaluating naïve patients treated with an empirical first-line quadruple therapy—either with or without bismuth—and excluding unaccepted and suboptimal triple therapies and tailored treatment based on the CYP2C19 polymorphism were included.

**Figure 4:**
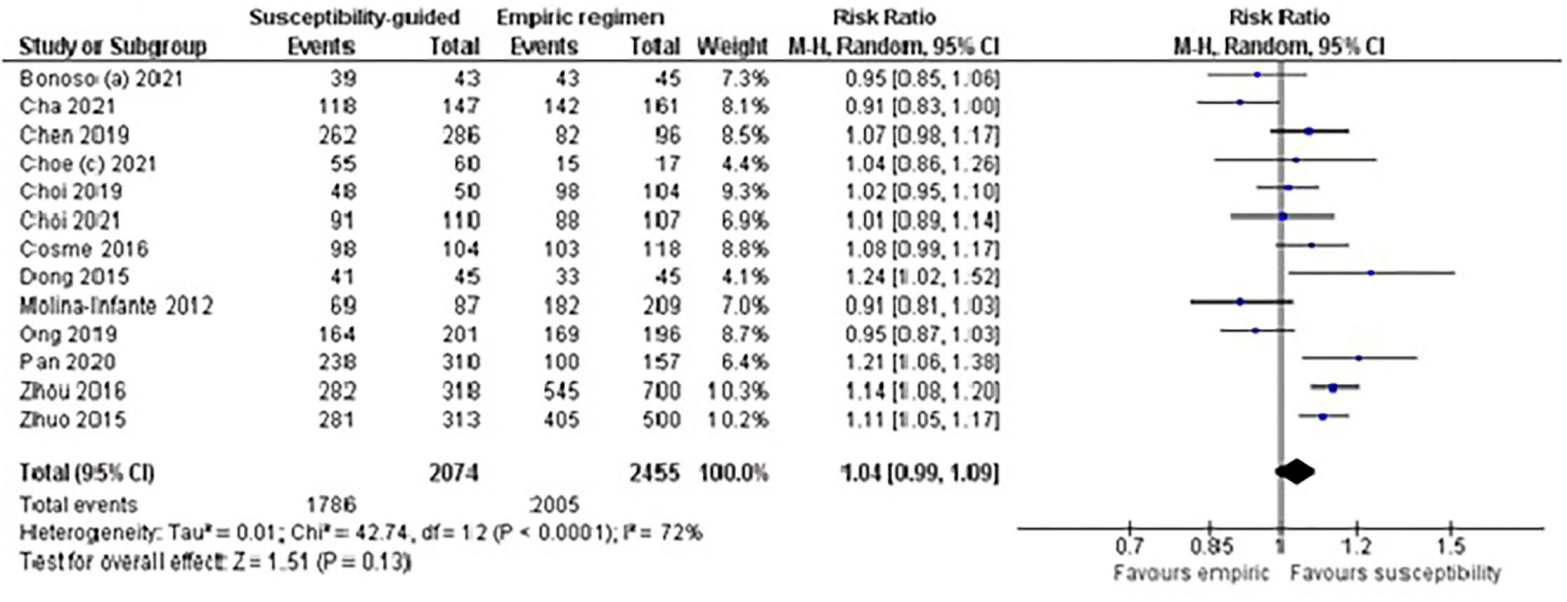
Forest plot of studies comparing the effectiveness of susceptibility-guided and empirical treatment in naïve patients treated with a (bismuth or non-bismuth) quadruple therapy. M-H, Mantel–Haenszel; CI, confidence interval.

In total, 12 studies (RCTs and non-RCTs) including 2,762 naïve patients (1,455 in the susceptibility-guided and 1,307 in the empirical group) were evaluated. No statistically significant differences were found in cure rates between the guided therapy (87%) and the empirical treatment (78%), with low to moderate heterogeneity between treatment arms (RR: 1.04; 95% CI: 0.99–1.09; *I*^2^: 72%; Figure 4). A *post hoc* sensitivity analysis including only RCTs (eight studies) in this same subgroup meta-analysis also confirmed the lack of difference in effectiveness between both groups (RR: 1.05; 95% CI: 0.99–1.12; *I*^2^: 77%). Moreover, excluding the RCT by Zhou et al. (2016), because the susceptibility-guided treatment was based on the CYP2C19 polymorphism, the effectiveness results remained similar in both treatment arms, with no significant differences (RR: 1.04; 95% CI: 0.97–1.12; *I*^2^: 75%).

### Quality Assessment

A summary of the quality of included studies is shown in Supplementary Figures 5, 6.

Quality assessment of all studies included in the meta-analysis is presented in the summary table, following Cochrane instructions for evaluation of comparative studies (both RCTs and non-RCTs) with the RoB tool.

The RoB for both the randomization and allocation items was either unclear or high-risk in 50 to 75% of the studies (Supplementary Figure 5). The quality items related to the blinding of participants and personnel and to the outcome assessment are unlikely to affect the eradication outcome because *H. pylori* is an objective measurable endpoint. Therefore, these items were considered as introducing a low risk of bias, even for open-labeled studies.

All studies reported complete outcome data with no imbalance between arms in the patient's participation flow; therefore, no attrition bias was identified. Likewise, no selective reporting bias was detected [except for one study (Bontems et al., 2011)], as results of the primary endpoint were always correctly reported and data could be extracted (Supplementary Figure 6).

The funnel plot comparing the susceptibility-guided vs. empirical regimen groups of all included studies are shown in Supplementary Figure 7. This plot shows asymmetry suggesting a possible publication bias.

## Discussion

Susceptibility testing has been proposed for antibiotic stewardship, aiming to reduce unnecessary antibiotic prescriptions; theoretically, treatment of *H. pylori* infections should not be an exception (Dang and Graham, 2017). Furthermore, through the application of susceptibility testing before treatment, the development of antimicrobial resistance could be minimized (Arslan et al., 2017), as antibiotic resistance in the outpatient community is positively correlated with antibiotic use (Megraud et al., 2013). However, in the present study (meta-analysis), the benefit of susceptibility-guided treatment over empirical treatment of *H. pylori* infection could not be demonstrated.

Several meta-analyses have previously compared cure rates of susceptibility-guided vs. empirical therapy for *H. pylori* first-line treatment, but all of them had limitations. The first meta-analysis was published by Wenzhen et al. (2010) and was focused specifically on first-line treatment. It only included five RCTs and concluded that culture-guided triple therapy was more effective than standard triple therapy (which was the regimen prescribed in most studies at that time) for first-line treatment. The second meta-analysis was published by Lopez-Gongora et al. (2015) and concluded that, in first-line treatment (nine studies only), susceptibility-guided therapy was more efficacious than empirical 7- to 10-day triple therapy (which, again, was the generally prescribed treatment at that time). The third meta-analysis was published by Chen et al. (2016) (including also nine studies only), and, again, showed that first-line tailored therapy achieved higher eradication rates than empirical regimens. The fourth meta-analysis was published by Gingold-Belfer et al. (2021) (including 16 studies only), also focusing mainly on first-line treatment (as only three RCTs were on rescue treatment), and concluded that susceptibility-guided therapy was superior to first-line clarithromycin-based triple therapy only when clarithromycin resistance exceeded 20%.

In our meta-analysis, the most updated in the literature, we have included 47 comparative studies (involving 6,705 patients in the susceptibility-guided group and 7,895 in the empirical group and including both RCTs and non-RCTs). Therefore, this study presents the highest number of studies in each subgroup published so far. Furthermore, the subgroup analyses performed in the present meta-analysis were more comprehensive than those of previous systematic reviews, and our protocol established no language restrictions.

Overall better efficacy results were obtained with the susceptibility-guided strategy for first-line treatments (naïve patients, 35 studies), although the results were borderline statistically significant (RR: 1.13; 95% CI: 1.08, 1.17). However, when prescribing only empirical quadruple regimens—that is, excluding the suboptimal triple therapies—no differences in efficacy were found vs. the susceptibility-guided group; this lack of difference was confirmed when only RCTs were considered. Therefore, we may conclude that susceptibility-guided treatment of *H. pylori* infection is not better than empirical treatment in first-line if the most updated bismuth or non-bismuth quadruple regimens are empirically prescribed, in agreement with a previous study (Gingold-Belfer et al., 2021).

The results of our meta-analysis are in agreement with the well-known high effectiveness of bismuth quadruple therapy, even in patients with clarithromycin or metronidazole resistance. In particular, when a bismuth quadruple therapy [either with tetracycline (Choi et al., 2019) or with amoxicillin (Chen et al., 2019)] was empirically prescribed, the efficacy was similar to that obtained with the susceptibility-based strategy. As an example, in the study by Choi et al. (2019), the eradication rate with the empirical bismuth quadruple and the susceptibility-based therapy was 94 and 96%, respectively. An advantage of prescribing a bismuth-based quadruple therapy is that we do not need to worry about previous antibiotic use or antimicrobial resistance as the risk of having a tetracycline or amoxicillin-resistant strain is extremely low and metronidazole resistance has limited impact on the effectiveness of this regimen (Gisbert and Pajares, 2002; Gisbert, 2020). In addition, the results of our meta-analysis are also in agreement with the encouraging results that are generally obtained with the empirical use of non-bismuth quadruple concomitant therapy, even when single clarithromycin or metronidazole resistance is present (only dual clarithromycin and metronidazole resistance seems to jeopardize effectiveness with this regimen) (Gisbert and Calvet, 2011).

Some meta-analyses have compared *H. pylori* cure rates of susceptibility-guided therapies with those of empirical therapy specifically for second-line treatment. In the meta-analysis by Lopez-Gongora et al., only four RCTs assessing *H. pylori* second-line rescue therapies were included (Lopez-Gongora et al., 2015). Results were highly heterogeneous and no significant differences were found between susceptibility-guided and empirical strategies in terms of efficacy. The other meta-analysis, performed by Chen et al., also found no differences between tailored and empirical rescue regimens, although only three studies were included (Chen et al., 2016). Finally, in our updated meta-analysis, for rescue therapies (16 studies, mostly as second-line), similar efficacy results were demonstrated with the two strategies—tailored and empirical—both when all the comparative studies were included and when only RCTs were considered.

It has been frequently recommended that performing culture at first-line treatment or after a first eradication failure may not be necessary and therefore assessing *H. pylori* sensitivity to antibiotics in clinical practice may be suggested only after failure of the second treatment (O'Connor et al., 2000). However, previous meta-analyses could not find any RCT comparing cure rates of susceptibility-guided therapies vs. those of empirical third-line therapy (Lopez-Gongora et al., 2015). Another systematic review aimed to evaluate the effectiveness of susceptibility-guided therapy as third-line therapy (without comparing it with empirical treatment) (Puig et al., 2016): four observational studies were included (no comparative studies were found), and the pooled mean eradication rate with susceptibility-guided therapy was only 72%. Therefore, the authors concluded that cure rates with susceptibility-guided therapy were, at best, moderate (Puig et al., 2016). Similarly, a more recent meta-analysis identified up to three studies and one sub-study showing a third-line therapy success of 79.9% in the susceptibility-guided therapy group vs. 65.2% in the empirical one (Gingold-Belfer et al., 2021). In our meta-analysis, four studies (of which two were RCTs) evaluated this comparison in the scenario of third-line treatment (Huang et al., 2018; Liou et al., 2018; Bonoso Criado et al., 2021; Choe et al., 2021), reporting no differences between the empirical and the susceptibility-guided arms. Therefore, the evidence is in favor of susceptibility-guided therapy as rescue therapy is currently insufficient to recommend its use.

In routine clinical laboratories, the detection of *H. pylori* antimicrobial resistance is mainly based on phenotypic methods performed after culture, including gradient diffusion susceptibility testing (E-test) and the agar dilution method (Arslan et al., 2017). In the last years, different PCR-based approaches have been developed as alternative tools to bacterium culture (Ierardi et al., 2017). In our meta-analysis, similar results were observed when susceptibility testing was assessed by culture or by PCR. Molecular tests are accurate in finding even minimal genotypic traces of certain resistant strains and are faster than conventional culture-based assays. Furthermore, PCR is technically feasible for clinical application in small- and medium-sized hospitals in developing countries (Xuan et al., 2016). However, the correlation between both methods is not perfect, probably due to the relatively low sensitivity of phenotypic assessment, the possibility that the E-test may identify resistant strains with point mutations different from those tested by PCR, or its inability to detect hetero-resistance (Ierardi et al., 2017; Jung et al., 2018).

Finally, some limitations of the strategy of performing culture systematically in all patients should be recognized: (1) culture implies the performance of endoscopic exploration, which is uncomfortable, expensive, and not free of risk. In addition, as endoscopy centers have been facing increasing demands, the technique frequently involves prolonged waiting times. As a consequence of the aforementioned problems, several diagnostic policies have been proposed for selecting patients with symptoms of dyspepsia, the most outstanding being the so-called “test-and-treat” strategy. Several prospective studies and decision analyses support the use of the test-and-treat strategy for dyspeptic patients (Gisbert and Calvet, 2013; Beresniak et al., 2020). Accordingly, this strategy has been recommended by all international consensus conferences (Fallone et al., 2016; Chey et al., 2017; Malfertheiner et al., 2017). (2) Culture is not always available on a routine basis. (3) The sensitivity of bacterial culture is not 100% (Megraud et al., 1997); indeed, even in the optimal conditions usually encountered in therapeutic trials, culture sensitivity is <90% (Zullo et al., 2003; Cammarota et al., 2014; Baylina et al., 2019). (4) Antibiotic susceptibility testing in clinical practice yields useful information only for a few antibiotics: clarithromycin, quinolones, and, less clearly, metronidazole (the relevance of *in vitro* metronidazole resistance for the *in vivo* treatment is quite limited) (Gisbert and Pajares, 2002); on the other hand, resistance to amoxicillin and tetracycline is extremely low. (5) Even knowing the susceptibility of *H. pylori*, eradication rates do not achieve 100%, as the results observed *in vivo* by following *in vitro* susceptibility to antibiotics are often disappointing (Guslandi, 2001; Gisbert and Pajares, 2002; Zullo et al., 2003; Baylina et al., 2019). The reverse situation is also possible, as *H. pylori* eradication may, nonetheless, be achieved in the presence of *H. pylori* metronidazole- or clarithromycin-resistant strains even with a drug combination including these antibiotics (Zullo et al., 2003; Bujanda et al., 2020, 2021). Furthermore, probably due to the synergistic effect of bismuth, the addition of this drug to triple therapy with clarithromycin may allow achieving a cure rate of approximately 90% even in patients with resistance against this antibiotic (Gisbert and McNicholl, 2017; Gisbert and Nyssen, 2021). (6) As previously mentioned, high eradication rates (≥90%) have been obtained with current up-to-date empirical first-line treatments, such as the bismuth or non-bismuth quadruple regimens. (7) Some studies have evaluated different empirical regimens after the failure of one, two, or more eradication treatments and have achieved a final (overall) eradication rate of almost 100% (Bock et al., 2000; Chan et al., 2000; Gasbarrini et al., 2000; Gomollon et al., 2000; Perri et al., 2000; Seppala et al., 2000; Beales, 2001; Canducci et al., 2001; Zullo et al., 2001, 2003; Treiber et al., 2002; Dore et al., 2003; Gisbert et al., 2003, 2004, 2008; Rokkas et al., 2009; Burgos-Santamaria et al., 2019). Thus, the empirical strategy should be based on the avoidance of repeating similar eradicating schemes, mainly clarithromycin- and quinolone-containing regimens, in the same patients during different eradicating regimens (Gisbert and Pajares, 2002; Roccarina et al., 2012; Calvet, 2018; Baylina et al., 2019; Nyssen et al., 2022). (8) Finally, different cost-effectiveness studies of the susceptibility-guided treatment of *H. pylori* infection have achieved contradictory results (Breuer and Graham, 1999; Romano et al., 2003; Qasim et al., 2004; Faber et al., 2005; Furuta et al., 2007; Cosme et al., 2013; Cammarota et al., 2014; Gweon et al., 2018; Liou et al., 2018; Cho et al., 2019).

Some relevant limitations affect studies comparing empirical vs. susceptibility-guided strategies, and consequently also the reliability of our meta-analysis. A major limitation of the current evidence regarding susceptibility-guided therapy is that comparative studies of susceptibility-guided therapy randomized patients after diagnostic endoscopy or even after successful culture (Lopez-Gongora et al., 2015). Therefore, the comparative effectiveness of susceptibility-guided therapy vs. the current non-invasive diagnosis and empirical treatment policy in patients with *suspected H. pylori* infection has not been evaluated in RCTs (Lopez-Gongora et al., 2015). Thus, a study adequately evaluating the effectiveness of susceptibility-guided therapy as a first-line treatment should randomize patients with non-investigated dyspepsia into non-invasive testing and endoscopy plus culture groups. In this same line, most of the studies evaluate the effectiveness of susceptibility-guided therapy as rescue therapy included the patients when the culture had been already obtained. Therefore, the effectiveness of susceptibility-guided therapy and empirical rescue therapy has never been properly compared (Puig et al., 2016). On the other hand, most studies using susceptibility-guided therapy only include patients with a positive culture. Therefore, the number of susceptibility-guided therapy failures due to patients' refusal of endoscopy has not been estimated or included (Baylina et al., 2019). When the applicability and effectiveness of this strategy were reviewed (Baylina et al., 2019), the rate of acceptance of endoscopy for biopsy and culture was described only in one article with only 60 patients and was reported to be as low as 60% (Matsumoto et al., 2005). In addition, given the diversity of studies included, our meta-analysis showed considerable heterogeneity (with asymmetric funnel plots) of the different *a priori* subgroup analyses performed comparing both therapeutic strategies; although such variability was investigated, it only could be partially explained. However, it is important to highlight that overall methodological quality was frequently high, and most studies were likely to avoid performance or detection biases (as per the therapeutic context) as well as attrition or reporting biases (as per the robustness of the outcome).

In summary, we think that susceptibility tests (culture or PCR) should be routinely performed, even before prescribing first-line treatment, in specialized centers with an interest in *H. pylori* management, to evaluate the prevalence of antibiotic resistance in the treatment of naïve patients and the influence of such resistances on the efficacy of up-to-date first-line eradication treatments. However, the present meta-analysis shows that the evidence is too limited to support the generalized use of susceptibility-guided therapy for *H. pylori* treatment in routine clinical practice, either as first-line or as rescue treatment. Undoubtedly, the most effective first-line *H. pylori* eradication treatment—that is, those regimens that have demonstrated to achieve cure rates ≥90% in our setting—must always be prescribed and the rescue treatment should be carefully chosen depending on which treatment was used initially. The results (*H. pylori* cure rates) of our clinical practice should be continuously audited to confirm that we always maintain a high success rate.

## Data Availability Statement

The original contributions presented in the study are included in the article/Supplementary Material, further inquiries can be directed to the corresponding author/s.

## Ethics Statement

Ethical review and approval was not required for the study on human participants in accordance with the local legislation and institutional requirements. Written informed consent from the participants' legal guardian/next of kin was not required to participate in this study in accordance with the national legislation and the institutional requirements.

## Author Contributions

OPN and JPG interpreted the data. OPN performed the analysis and prepared the first draft of the manuscript. ME, JPG, and OPN contributed to the literature search and data extraction. ME reviewed and approved the final draft of the manuscript. JPG analyzed and reviewed the draft manuscript. All authors contributed to the article and approved the submitted version.

## Conflict of Interest

JPG has served as a speaker, consultant, and advisory member for or has received research funding from Mayoly, Allergan, Diasorin, Gebro Pharma, and Richen. OPN has received research funding from Mayoly and Allergan. The remaining author declares that the research was conducted in the absence of any commercial or financial relationships that could be construed as a potential conflict of interest.

## Publisher's Note

All claims expressed in this article are solely those of the authors and do not necessarily represent those of their affiliated organizations, or those of the publisher, the editors and the reviewers. Any product that may be evaluated in this article, or claim that may be made by its manufacturer, is not guaranteed or endorsed by the publisher.

## Abbreviations

*H. pylori*: *Helicobacter pylori*
MIC: minimal inhibitory concentration
PPI: proton pump inhibitor
RCT: randomized controlled trial.
