# Supplementary figures and images for: Empirical vs. Susceptibility-Guided Treatment of Helicobacter pylori Infection: A Systematic Review and Meta-Analysis
Source: Front Microbiol. 2022 Jun 14;13:913436. doi: 10.3389/fmicb.2022.913436 (PMC9237546; doi:10.3389/fmicb.2022.913436)

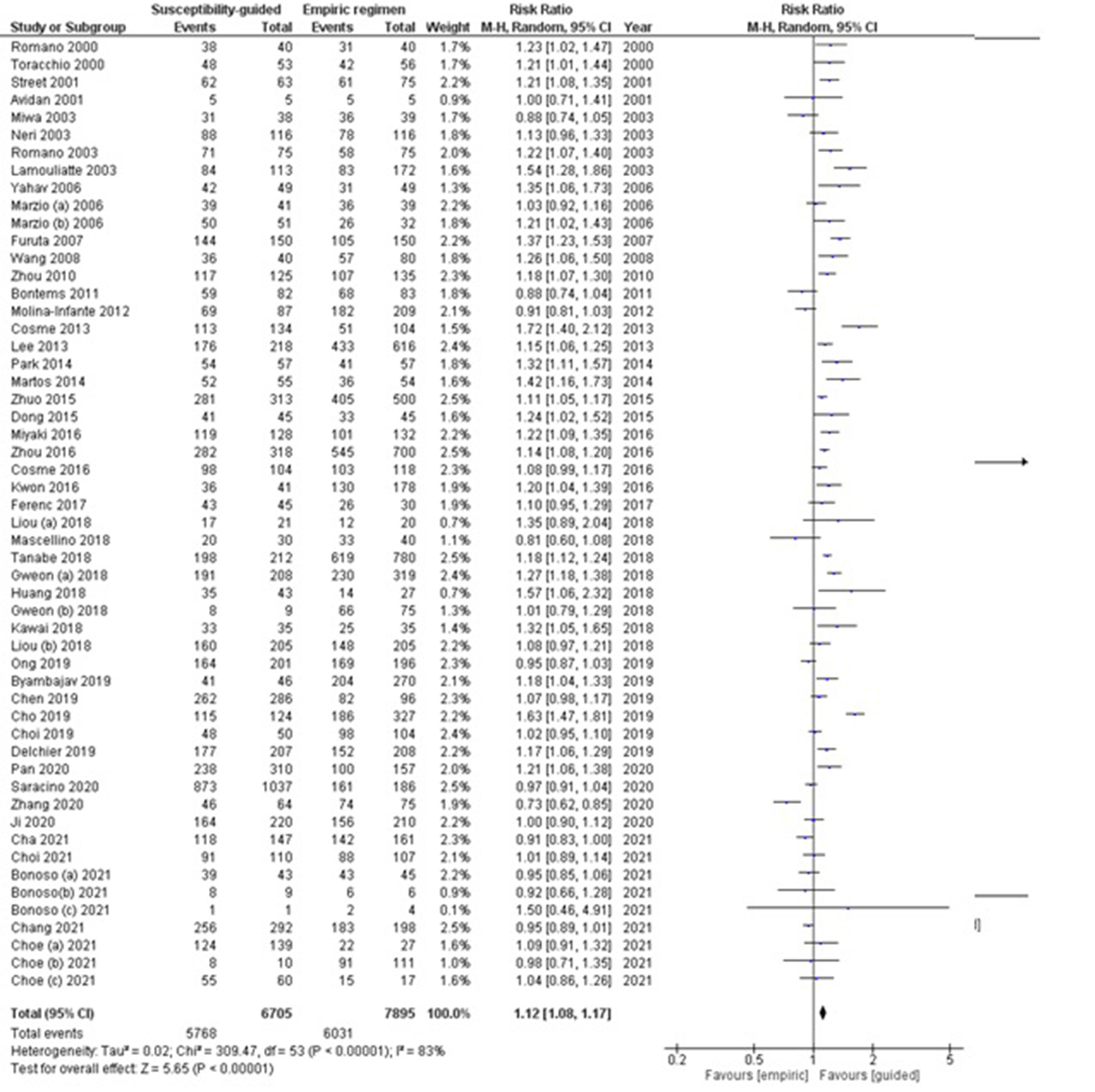

Supplement: Supplementary file 1 [file Image_1.JPEG]

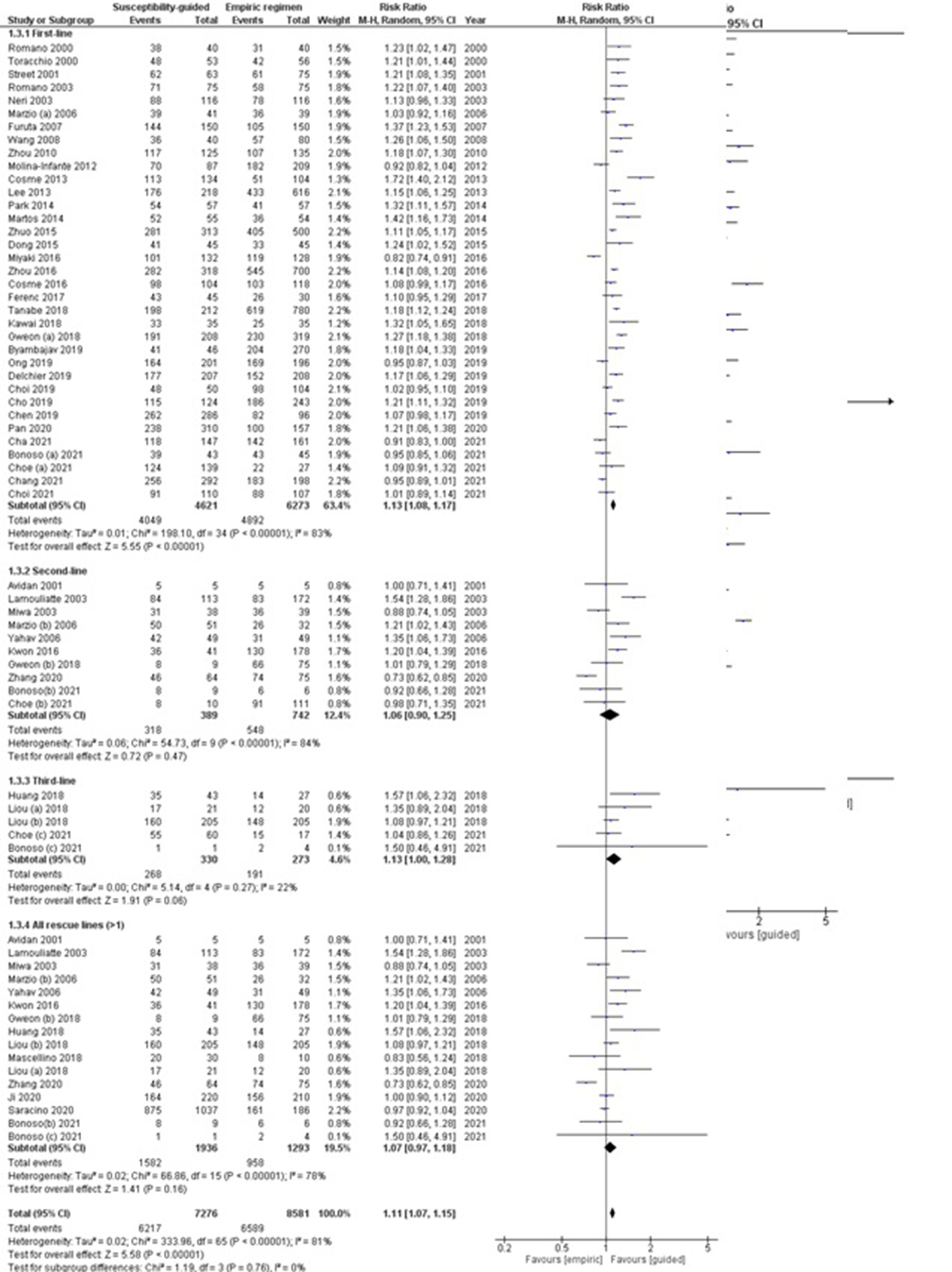

Supplement: Supplementary file 2 [file Image_2.JPEG]

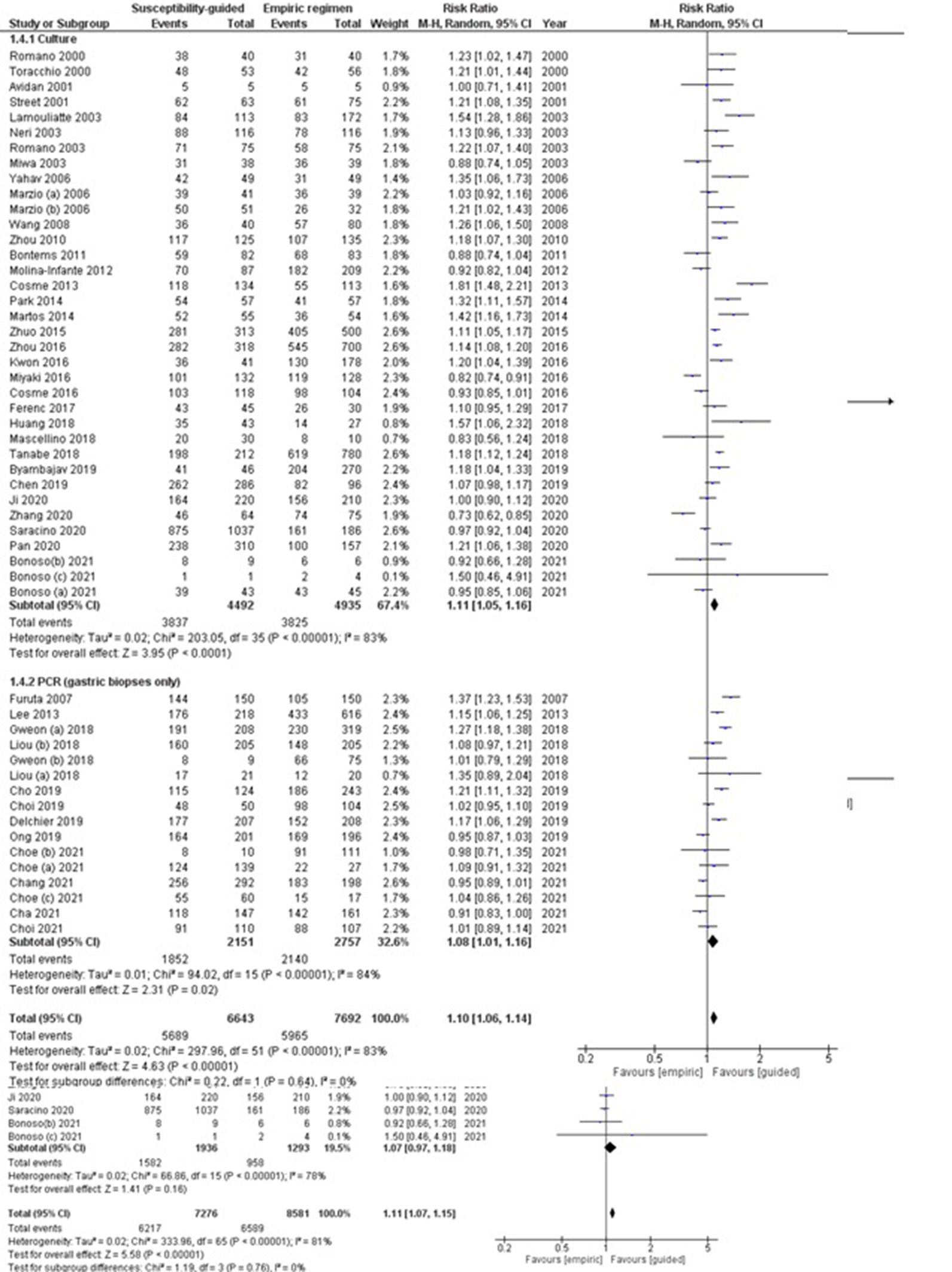

Supplement: Supplementary file 3 [file Image_3.JPEG]

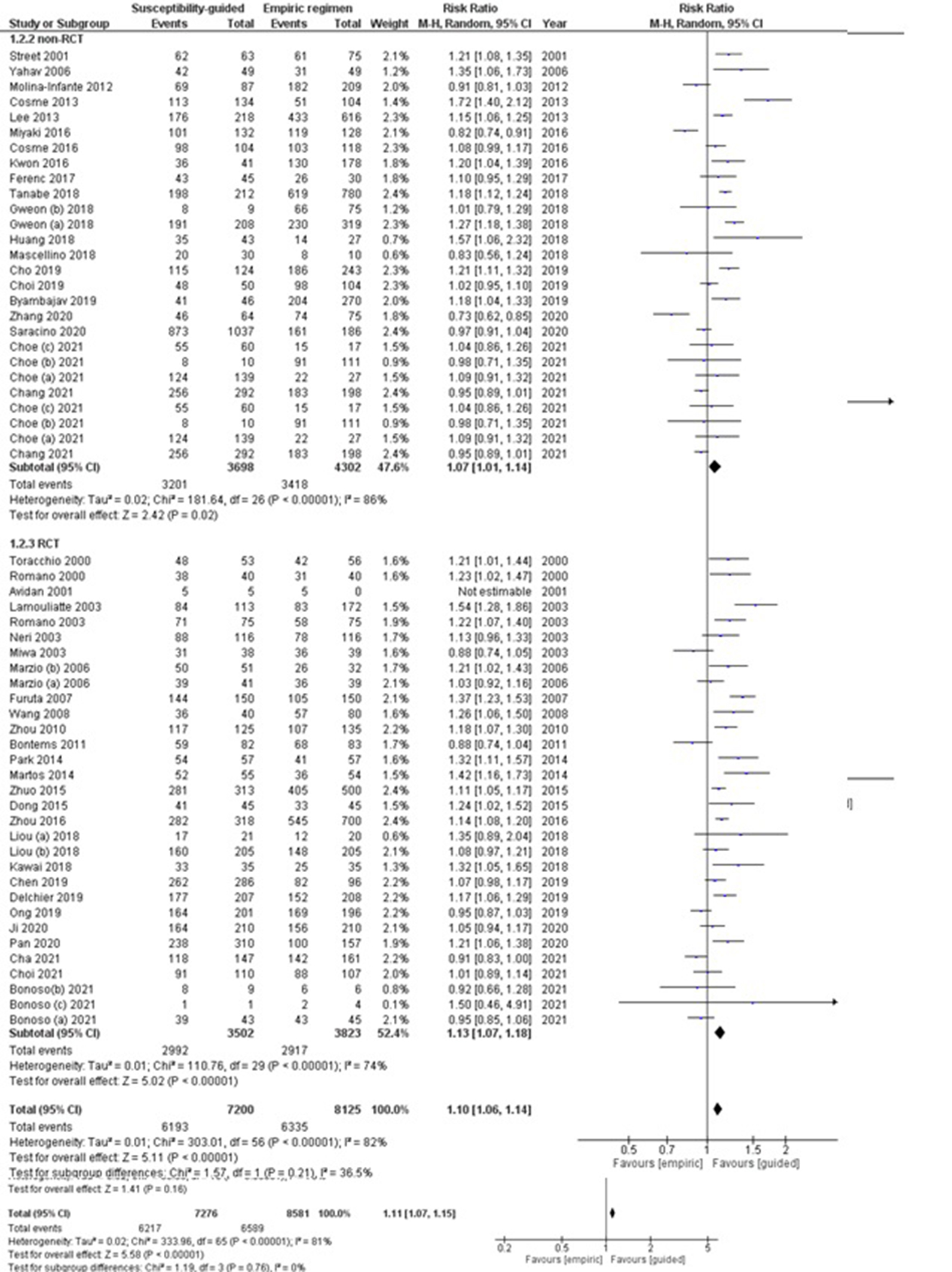

Supplement: Supplementary file 4 [file Image_4.JPEG]

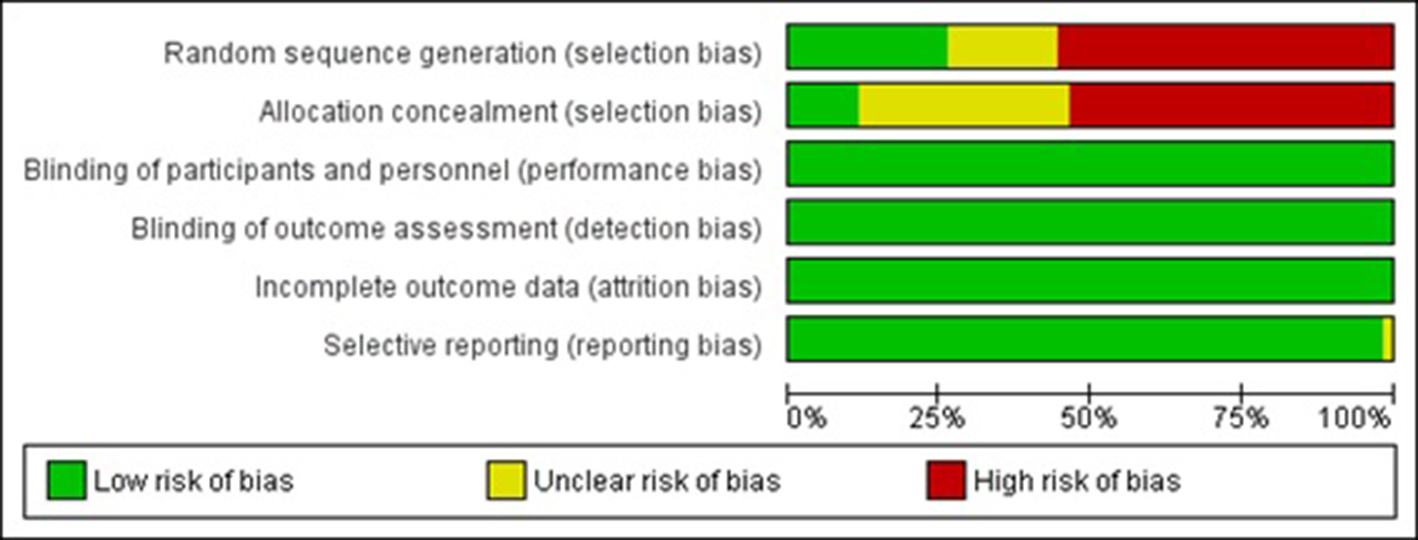

Supplement: Supplementary file 5 [file Image_5.JPEG]

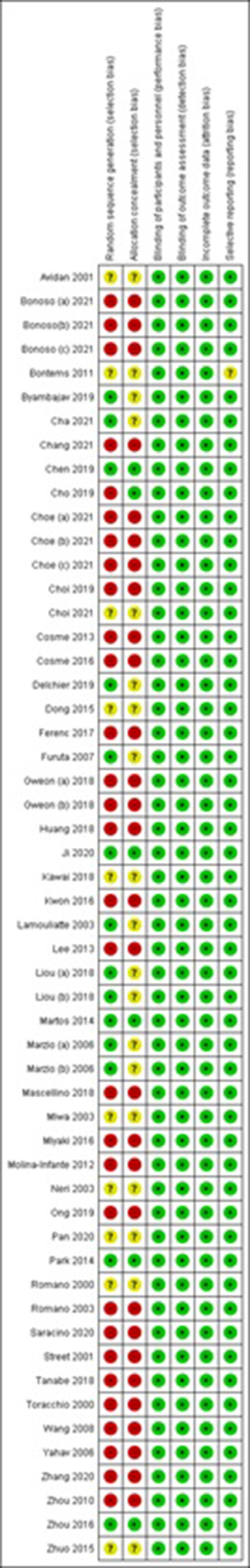

Supplement: Supplementary file 6 [file Image_6.JPEG]

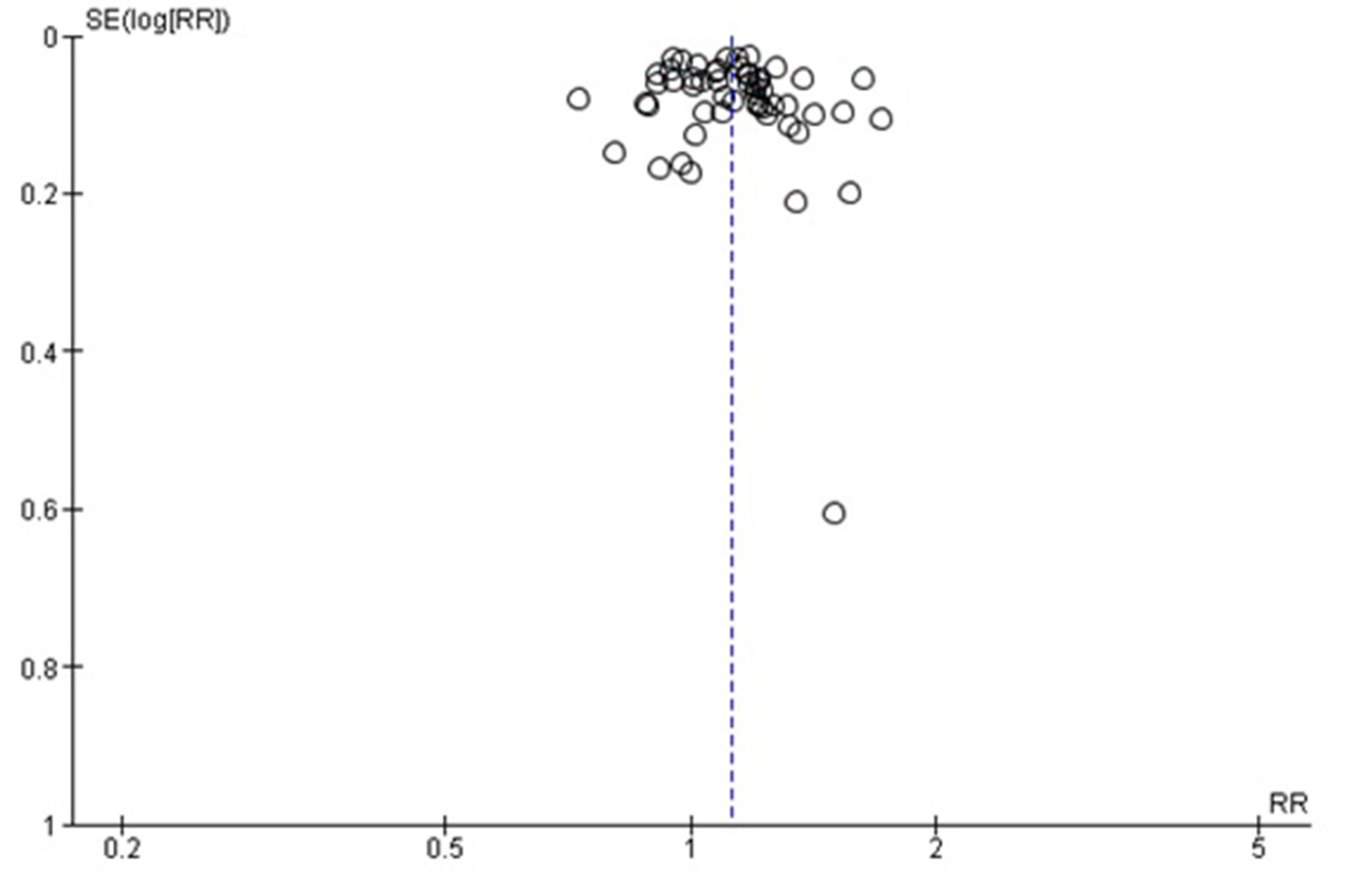

Supplement: Supplementary file 7 [file Image_7.JPEG]
